# Supplementary material for: Are carbon emissions trading and green financial instruments synergistic? -Comprehensive quantitative research based on content analysis
Source: PLoS One. 2024 Mar 7;19(3):e0298601. doi: 10.1371/journal.pone.0298601 (PMC10919601; doi:10.1371/journal.pone.0298601)
Supplement: S1 Data — (ZIP) [file pone.0298601.s001.zip › Data/地级市Z_F工作报告.zip!/╣π╢1⁄2/╢1⁄2▌╕╩╨/└┤╫╘╣1⁄2╓┌║┼í╛┬φ┐╦╩2╛▌═°í┐_2017.docx]

各位代表：

我代表市人民政府，向大会报告政府工作，请予审议，并请市政协委员和其他列席人员提出意见。

过去五年回顾及2016年主要工作

市第十五届人民代表大会第一次会议以来的五年，是我市加快转型升级、建设幸福东莞、实现高水平崛起的五年，是东莞加快建设国际制造名城、现代生态都市的五年。五年来，面对严峻复杂的宏观形势和改革发展稳定的艰巨任务，全市政府系统和社会各界一道，在上级和市委的坚强领导下，强化“三个走在前列”的使命担当，凝心聚力，砥砺奋进，较好地完成了市十五届人大历次会议确定的目标任务。

这五年，是东莞主动适应经济发展新常态、推动高水平崛起、综合实力稳步提升的五年。紧紧扭住发展第一要务，积极应对经济下行压力，全力推动东莞经济行稳致远。经济实力稳中有进。全市生产总值相继突破5000亿元和6000亿元，预计达到6770亿元，比2011年增长46.6%，年均增长8%。来源于东莞的财政总收入、市一般公共预算收入、金融机构各项存款余额分别达1569亿元、545亿元和11500亿元，是五年前的1.87倍、1.74倍和1.77倍。三次产业比例从0.4：50.7：48.9调整为0.3：46.5：53.2。东莞经济总量名列全国大中城市第21位。在中国社科院与联合国人居署联合发布的报告中，东莞城市竞争力排全球第154位、全国第10位（含港澳台）、广东第3位。园区实力持续提升。松山湖纳入珠三角国家自主创新示范区，在全国国家高新区综合排名由第53位升至第29位。东莞港吞吐量从58万标箱增长到350万标箱，成为全国第十一大沿海港口、珠三角第二大内贸港口。松山湖与生态园、沙田镇与东莞港、长安镇与滨海湾开发区统筹整合，水乡经济区、大学创新城、银瓶创新区等“三大增长极”初具雏形。镇村实力继续壮大。镇街平均生产总值突破200亿元。300亿元以上镇街由1个增至7个，3个镇街进入400亿元俱乐部。村组两级总资产和经营纯收入分别达到1508亿元和124亿元，比2011年增长22.2%和57%；资产负债率17.4%，五年下降5.3个百分点。企业实力稳步增强。“三重”建设扎实推进，五年来累计引进重大产业项目268宗、完成投资1015亿元。全市市场主体增至84万户。主营收入1000亿元企业实现零的突破，500亿元、100亿元、50亿元企业分别增至3家、11家和31家，超10亿元企业数位居全省地级市首位，拥有境内外上市企业33家。新三板挂牌企业168家，总量居全省地级市首位、全国地级市第三。“星月同辉”的格局逐步形成。城市影响力不断提升。东莞先后实现全国文明城市“三连冠”、全国双拥模范城“八连冠”，蝉联全国创新社会治理优秀城市、全国社会治安综合治理优秀市，荣获全国质量强市示范城市、国家知识产权示范城市、国家电子商务示范城市、国家森林城市、国家节能减排财政政策综合示范城市、国家公共文化服务体系示范区等称号。

这五年，是东莞打造创新型经济、转型升级步伐加快、发展质量效益提高的五年。深入实施创新驱动发展战略，科技金融产业融合步伐加快，产业转型升级取得扎实成效。创新能力有效提升。全市R&D比重预计达2.4%，增速连续五年排全省第一位。国家高新技术企业从413家增加到1500家，省级创新科研团队从9个增加到26个，总数均居全省地级市首位。新增新型研发机构17个、科技孵化器42个、博士后科研工作平台44个，总数分别达到32个、48个和68个。制造业转型升级加快。率先对接国家战略实施“东莞制造2025”战略，智能制造等“六大工程”全面推进，“机器换人”深入实施。先进制造业、高技术制造业增加值分别占规模以上工业增加值的48.5%和38%，比2011年提高6.6和9.3个百分点。服装、家具、五金模具等传统产业加速向“微笑”曲线两端延伸。新增全国知名品牌创建示范区2个。省级专业镇达34个。省级以上企业工程（技术）中心、名牌名标达到247个和558个，分别增长165.6%和62.7%。“四新”经济蓬勃发展。智能手机产业成为经济新增长点，2016年出货量达到2.55亿台，约占全球20%。机器人及智能装备、电子商务、现代物流、文化创意等产业加快发展，为产业升级注入了新动力。内外源经济协调发展。预计全市进出口总额、出口总额分别达11000亿元和6500亿元，稳居全国大中城市第5位和第4位。“一带一路”战略扎实推进。加工贸易自主性、根植性增强。外资企业新设立研发机构1265个，总数达到1712个。规模以上工业企业内销比重超过外销，民营工业占比提高17.8个百分点，民营经济税收与增加值贡献率逐年提高。

这五年，是东莞推进重要领域改革、体制机制活力增强、各项改革全面深化的五年。稳步推进国家和省50多项改革试点任务，全面推进330多项莞版改革措施，努力向改革要动力要红利，打造法治化国际化营商环境。行政审批改革深化提速。项目审批流程再造和直接落地改革成效凸显。水陆口岸“三互”大通关改革减少企业一半以上手续和时间。“放管服”改革积极推进。五年精简行政审批项目512项，向镇街（园区）下放经济社会管理权限546项，复制推广自贸区改革政策63项。市场活力有效激发。商事制度改革全面铺开，东莞商改模式成为全国样本，企业注册时间平均缩短60%以上，市场主体比2012年改革前增加29.9万户，增长55.2%。五年取消、下调或免征停征涉企行政事业性收费260多项。通过各种途径累计为企业减负近280亿元。东莞营商环境“加一”、综合成本“减一”的努力得到广泛认可。与此同时，土地生态利用制度、农村综合改革、基层社会治理创新、社区网格化管理和微治理等一大批特色改革扎实推进，为经济社会发展带来了更多改革红利。

这五年，是东莞践行以人为本理念、城乡面貌不断改善、社会民生事业长足进步的五年。加快国家新型城镇化综合试点步伐，加强公共安全管理，推进幸福东莞建设，让人民群众享有更宜居的城市环境、更安定的社会环境、更优质的公共服务。城市功能日趋完善。东莞新火车站、地铁2号线、莞惠城际轨道常平至惠州段、沿江高速、博深高速等一批交通基础设施建成，市篮球中心、市民艺术中心等一批公共服务设施投入使用。生态环境持续优化。完成江库联网一期工程。茅洲河等跨界河和内河涌污染治理全面加强。空气质量明显改善。水乡地区“两高一低”企业加快退出。19.6万辆黄标车和老旧车顺利淘汰。推广应用新能源汽车2800辆。超额完成“十二五”节能减排任务，单位生产总值能耗降幅及二氧化硫、氮氧化物减排量位居全省第一。新建森林公园、湿地公园17个，建成绿道956公里、休闲绿地1287处。社会治理水平提升。社会建设全面加强，创建全省创新社会管理引领区成效明显。“黄赌毒”现象得到铁腕整治，规模化、组织化“涉黄”犯罪强力清除，“两抢一盗”等违法犯罪行为逐步下降，全市破案数上升34.2%，命案数压减32.7%。安全生产、食品药品安全等得到切实加强，劳资纠纷、群众信访等得到妥善处置，市民法治意识显著增强，社会文明程度持续提高。社会民生事业全面发展。坚持每年办好十件实事。城乡居民人均可支配收入达到41782元，比2011年增加65.4%。文化名城战略扎实推进，公共文化服务体系日益完善，一大批艺术精品获国家级大奖，“音乐剧之都”的品牌更加响亮。连续五年提高基本养老金，城乡救助及福利水平逐步提高，在全国率先建立起城乡一体化的社会养老和医疗保险体系。成功创建广东省推进教育现代化先进市。体育、医疗、就业、住房保障、住房公积金、武装和民兵预备役等工作全面加强，基本公共服务均等化扎实推进，市内外对口帮扶任务进展顺利，群众在发展中得到更多实惠。

这五年，是东莞加强政府自身建设、依法行政全面推进、工作作风持续改进的五年。切实转变政府职能，加强民主法治和廉政建设，打造为民务实清廉政府。机关作风进一步优化。在政府系统相继开展群众路线教育实践活动和“三严三实”、“两学一做”等专题教育，撤并72.7%的考核检查评比表彰活动，压减47%的节庆、论坛、展会活动，“四风”问题、“不严不实”问题得到积极整改。政府职能进一步转变。物价、商贸、卫生、计生等职能调整理顺。编制市镇两级政府权责清单，开展行政审批标准化建设，梳理分类行政许可和公共服务事项1812项。市网上办事大厅初步建成，各镇街及园区办事站建设实现全覆盖，建成593个社区综合服务管理中心。12345政府服务热线开通。行政机关负责人出庭应诉制度不断完善。依法行政水平进一步提升。法治政府建设深入推进。东莞正式获得地方立法权，城管、环保、交通、文化等领域地方立法工作有序铺开。“六五”普法顺利完成。在全省率先实现一村（社区）一法律顾问全覆盖。主动接受人大法律监督、政协民主监督和社会舆论监督。在中国政法大学近年发布的法治政府评估报告中，东莞总分均位居全国地级市前列。

刚刚过去的2016年，是实施“十三五”规划的开局之年，全市政府系统认真贯彻落实上级和市委的决策部署，较好地完成了年度目标，205项具体任务高效落实，十件实事圆满完成，实现了“十三五”的良好开局。刚刚过去的2016年，也是东莞承前启后、深化改革发展的一年，全市政府系统按照市委的统一部署，以供给侧结构性改革为统领，以构建开放型经济新体制综合试点试验为契机，开展八大专题调研，摸清了现状，找准了短板，理清了思路，不少调研成果正逐步转化为指导政府工作开展的系统方案。李克强总理莅莞考察时，对东莞在动能转换过程中保持较快发展速度、经济结构和动能不断优化表示了肯定，指出这一走势很好地说明新旧动能正在加速转换，希望东莞保持发展势头，让新动能逐步挑起大梁，旧动能不断焕发生机。一年来，我们突出抓了以下工作：

——着力保障经济稳中向好，扎实推进供给侧结构性改革。预计全市生产总值增长8.1%左右，快于全国、全省增速。进出口总额增长10%左右，增速在全国五大进出口城市中位居第一，其中出口增长2%左右。东莞成为全国第2个国税突破1000亿元的地级市。落实“三去一降一补”。全年为企业减负超200亿元。产业供给结构进一步优化，先进制造业、高技术制造业增加值分别增长15.1%和17.8%。新增主营收入超10亿元企业17家，总数达243家。新增500亿元企业2家。华为终端率先突破千亿元。全力抓好重大项目建设。全年完成投资439.4亿元，超过计划18.7个百分点，同比增长10.5%，完成情况近年最好。总投资99亿元的19个重大产业项目建成投产。加快战略性新兴产业发展。智能手机主营收入近3000亿元，增长40%以上。华为、OPPO、VIVO手机出货量均进入全球前六、稳居全国前三。在智能终端产业的带动下，电子信息制造业规上工业增加值增长20%左右。全市新登记新兴产业市场主体6207户，增长113%。

——着力强化创新驱动，加快集聚创新要素。抓好高企培育“育苗造林”计划和孵化体系建设。全市新增高企761家、高企后备企业819家，总量均居全省地级市第一。用好相关政策，为高企减免税款32.3亿元，增长54.5%。新增新型研发机构6个、科技孵化器12个、国家级众创空间9个。促进科技金融产业紧密融合，新增上市企业2家、新三板挂牌企业105家。实施“机器换人”带动智能制造。全市申报“机器换人”项目577个，总投资72亿元，其中莞产设备占16%。项目数和总投资额均居全省第一，带动全市工业技改投资增长35%。加快国家自主创新示范区建设。松山湖生产总值、税收分别增长16%和24.3%，综合实力在全省国家高新区中排名第三。全国智能制造试点示范经验交流会、全省推进珠三角创新驱动发展培育高新技术企业工作现场会、专业镇协同创新工作现场会在莞召开，东莞相关经验得到肯定和推广。

——着力优化对企服务，促进外资和民营经济协调发展。高规格召开非公经济工作会议，制定实施提升外经贸水平45条和民营“亲企清政”36条。扎实推进外贸稳增长调结构。推动加工贸易企业提质增效，加快广东（石龙）铁路国际物流基地和中俄贸易产业园建设，成功举办海丝博览会、加博会、智博会、台博会等展会。对“一带一路”国家出口超过1350亿元。跨境电商贸易增长迅速，国际邮包出口量突破7000万件，跃居全国第四。国际邮件互换局兼交换站落户东莞。实施“亲企清政”工程。建成“千干扶千企”网络平台，开展“问暖企业总部”系列活动。民营规上工业增加值增长18.3%，占全市比重达38.6%。民间投资增长13%，占固定资产投资总额的70%。新登记非公经济市场主体17.1万户，增长41%。毫不松懈加强招商引资。主动加强与周边城市产业合作。组团赴欧美、日韩及全国各地招商。全市合同外资47.3亿美元、实际外资39.3亿美元，扣除不可比因素分别增长8.8%和3.1%。协议内资1174亿元。实际内资662亿元，增长20%。

——着力促进区域协作，积极推动优势互补、密切合作。主动对接广东自贸区，深莞惠3+2经济圈合作日益紧密，莞深产业合作不断强化。实施新一轮市内帮扶工作，落实帮扶专项资金1.8亿元，支持次发达村加快发展。对口帮扶韶关工作在全省考核中排名第三。推进韶关、揭阳精准扶贫精准脱贫工作，完成到村帮扶项目1026个，到户项目36218个。启动对口帮扶云南昭通工作。援疆工作进展顺利，兵团草湖广东纺织产业园首期30万锭项目建成投产。援藏援川工作成效明显。

——着力优化城市环境，加速推进重大基础设施和生态环境建设。加快轨道交通等建设。地铁2号线开通运营，累计客流量2160多万人次。莞惠城际轨道常平至惠州段通车。启动地铁1号线工程前期工作。与深圳、广州加快研究地铁对接规划。从莞高速主线、粤晖大桥等项目建成。虎门二桥、深圳外环高速东莞段、莞番高速扎实推进。打通了南丫大桥等5条断头路。实施信息基础建设大会战和电网大会战，WIFI接入点建设规模全省第一，建成110千伏及以上电网工程项目15个。深入推进污水和固废治理。全面打响新一轮水污染治理攻坚战。加强茅洲河、石马河污染整治。建成260公里截污管网，完成4家污水处理厂扩建，新增污水处理能力17万吨/日。试点推进麻涌、清溪的分散式污水处理项目建设。综合整治桥头大东洲等3个生活垃圾填埋场。完成厚街环保热电厂二期扩建工程，一批环保热电厂扩建工程扎实推进。农村生活垃圾处理连续两年全省考核第一。抓好节能减排和大气污染治理。全面完成国家节能减排财政政策综合示范城市建设任务，推动水乡地区101家污染企业整治和退出，主要污染物排放量持续下降。单位地区生产总值能耗下降5%以上。空气优良天数达到318天，同比增加11天。

——着力开展文明创建，全面实施“四大提升工程”和“十大专项行动”。深入开展城市文明“补短板促提升”工作，集中整治户外广告、城市“牛皮癣”、环境卫生、交通秩序，大力实施核心价值观融入提升、市民素质提升等行动，广泛开展“东莞好人”评选活动，着力打造“友善之城”，市、镇、村一体化文明创建取得阶段性成效。全市增设核心价值观等公益广告17.3万块，公益广告比例达到30%以上。实施文明创建工程项目311个、总投资额超过9.5亿元。镇容村貌更加整洁有序，清理卫生死角超过52万处、关停处理“牛皮癣”号码近1.3万个，签约“门前三包”责任书150多万份。城市精细化管理进一步加强，处理占道经营超过33万宗、违章建筑超过1万处。城市品位和城市形象有效提升。

——着力维护安定和谐，以“智网工程”等为抓手提升社会服务管理水平。全面启动“智网工程”。划分基础网格3201个，配置管理员9177人，首批推动公安等七个部门77个事项入格，在所有镇街（园区）全面铺开。全市实有人口公共基础信息平台上线试运行。努力提升群众安全感。深入开展“飓风2016”等专项行动，严厉打击各类突出违法犯罪，强化命案防范打击，开展“以案说防”活动，提升“全民创安”参与度。狠抓安全生产、消防和信访工作。严格履行安全生产责任，深入开展危险化学品和易燃易爆物品安全、火灾隐患重点地区“百村挂牌”督办、“三小”场所和出租屋消防安全、建筑施工安全等领域的专项整治，整改各类风险隐患20多万处。加强信访源头防控和积案化解，妥善处置劳资纠纷、非法集资等引发的群体性事件，社会大局保持和谐稳定。

——着力加强民生保障，全面发展文教体卫等社会事业。实施文化惠民工程。推进国家公共文化服务标准化、基层综合性文化服务中心和国家数字文化馆试点建设。开展“寻找最美家庭”活动。加强文艺精品创作。推出首部本土题材音乐剧《虎门销烟》，完成大型电视剧《袁崇焕》现场拍摄。提升教育公共服务水平。注重民办教育发展，新设6.5亿元民办教育专项资金，新创建义务教育阶段优质民办学校39所。重视解决随迁子女教育问题，铺开向民办学校购买学位政策，为随迁子女提供学位3.5万个，增长23.8%。在22所学校实施托管改革，推进莞式“慕课”教学试点。高考各项指标位居全省前列。东莞被认定为全国义务教育发展基本均衡市。扎实推动卫生、体育等社会事业发展。推进公立医院医疗服务价格改革，推行分级诊疗制度，方便群众就医。对8家市属公立医院给予2.05亿元补助，减轻医院负担。基本医疗保险年度最高支付限额由20万元增加到30万元。有序实施“全面两孩”政策。建立危重症孕产妇和新生儿急救网络。市社会福利中心改扩建项目投入使用。发放就业创业补贴3.68亿元。成功举办国际马拉松赛和亚欧男子乒乓球全明星对抗赛。出台住房限购政策，促进房地产市场平稳健康发展。认真做好台风“妮妲”、“海马”防御工作。国防动员、统计审计、外事侨务、工青妇幼、民族宗教、档案方志、残疾人、红十字会、打私等工作有效推进。在2016中国地级市民生发展100强城市中，东莞名列第3位。

各位代表，过去五年，我们攻坚克难，锐意进取，迈出了转型升级的新步伐，谱写了改革发展的新篇章。这离不开上级和市委的正确领导，离不开历届党委政府打下的坚实基础，是全市人民共同奋斗的结果。在此，我代表东莞市人民政府，向全市干部群众，向人大代表和政协委员，向各民主党派、各人民团体、社会各界人士，向各驻莞单位、驻莞部队和武警官兵，向所有参与和支持东莞建设发展的港澳台同胞、海外侨胞和国际友人，表示衷心的感谢和崇高的敬意！

与此同时，我们也清醒地看到，全市发展仍然存在不少困难和问题，需要引起我们高度重视：一是经济下行压力仍然较大。外部市场环境复杂严峻，高端制造回流和低端市场分流同时并存，引进的重大项目建成释放产能尚需时日，新的经济增长点仍较单一，稳增长的基础有待加强。二是新旧动能转换还处于胶着状态。传统的路径依赖尚未根本转变，集约发展水平有待提高，扩大优质供给和有效需求难度较大，一些企业和行业仍缺乏核心竞争力，转型升级任重道远。三是利益固化的藩篱制约了深层次改革的推进。随着改革步入深水区，一些深层次矛盾和长期积累问题亟待攻坚破解。进一步解放思想的观念束缚、局部碎片化的土地利用状况、固化胶着的利益格局等，制约了全市改革向纵深推进，统筹发展、征地拆迁、城市更新、“两违”整治等工作仍需加大力度突破。四是环境污染治理任务艰巨。大气、土壤等污染治理以及垃圾处理问题亟待解决，特别是水环境整治历史欠账较多，深度治理刻不容缓。五是城市品质和内涵有待进一步提升。与优质企业和高端人才的需求相比，基础设施和城市配套还需完善，精细化管理需要进一步加强，化解社会矛盾和消除安全隐患的能力有待提升，文化、教育、医疗、交通等公共服务仍待优化。六是作风建设和履职水平仍需改进。一些干部存在“顺其自然”的工作心态，攻坚克难的锐气和韧劲不够，甚至消极对待、无所作为，影响工作推进。对此，我们必须高度警醒，尽最大努力予以解决。

今后五年的奋斗目标和主要任务

今后五年，是东莞加快经济转型、实现动能转换的决胜时期，也是我市加速城市升级、促进和谐善治的关键时期。市第十四次党代会明确了“在更高起点上实现更高水平发展”的价值追求，提出了全市发展的主要目标：一是率先全面建成小康社会，率先迈上基本实现社会主义现代化新征程；二是跨越生产总值万亿元；三是跨越“中等收入陷阱”；四是动能转换与经济转型取得根本性突破；五是社会和谐善治水平得到根本性提升。发展蓝图已经绘就，全市政府系统必须紧紧围绕市委决策部署，紧密结合东莞实际，以清晰的思路、有力的措施抓好工作落实，促进全市经济结构更优、发展动力更足、体制机制更活、城市环境更美、人民生活更好，奋力谱写东莞改革发展的新篇章。

今后五年，要在更高起点上实现更高水平发展，必须练好内功，率先突围，促进城市综合实力再上新台阶。从当前态势综合判断，今后几年总需求低迷和产能过剩并存的格局难以根本改变。国家正大力推进供给侧结构性改革，主动摆脱“粗放发展”，努力实现“内涵增长”。实践证明，这是适应新常态、应对下行压力的最佳途径。无论对一个企业、还是一座城市，今后几年都是练好内功、加快转型、提升核心竞争力的关键时期。我们必须保持定力，坚定转型，毫不动摇地强化创新驱动发展，加快转型升级步伐，切实优化产业供给结构，不断增强东莞先进制造业的核心竞争力；必须造好环境、打好基础，以提升城市品质和内涵为核心，沉下心来，更高水平地推进新一轮规划建设，加强精细化管理，增强综合承载力，把东莞打造成创新创业热土和宜居生态城市。力争未来五年，随着转型成效的逐步显现和新动能的逐步释放，全市地区生产总值年均增速达到8.3%以上，到2021年超过万亿元，东莞的综合实力与城市地位进一步提升，继续处在全国地级市前列。

今后五年，要在更高起点上实现更高水平发展，必须以最积极主动的态度集聚高端资源，强化创新驱动，实现新旧动能的转换和转型升级的突破。随着粤港澳大湾区一体化进程的加快，珠三角城市群正处于区域分工格局调整的关键时期。周边中心城市作为产业高地和创新中心，辐射带动能力日益增强。东莞依托优越的区位优势和坚实的产业集群优势，正日益成为高端产业加速和产能扩张的首选之地，迎来了新一轮产业大发展的重大历史机遇。我们必须正视港珠澳大桥、深中通道开通后发展格局的变化，以强烈的使命感和紧迫感，抓住这3—5年的窗口期，努力在新一轮产业发展中占据先机、赢得主动。在产业增量上，以最积极主动的态度，腾挪发展空间，狠抓招商引资，集聚高端资源，引进一批支撑城市未来发展的重大优质项目和先进产业集群。同时，推动已列入“十三五”规划、总投资5779亿元的317项重大项目落地，最快速度释放产能。在产业存量上，出台实施倍增计划，市镇两级集中力量扶持一批存量优势企业规模与效益倍增，形成市镇联动、以点带面的良好态势，促进全市产业总量扩张和集约发展。在产业动力上，深入实施创新驱动发展战略，加快建设珠三角国家自主创新示范区，培育高企2500家以上，新型研发机构50家以上，科技孵化器加速器100家以上，让东莞制造插上科技与金融两大翅膀。深入实施“东莞制造2025”战略，加快“机器换人”和智能化改造，不断提高东莞制造的自动化、智能化、绿色化水平。深入推进构建开放型经济新体制综合试点试验，促进传统产业加快转型、加工贸易创新发展、民营经济提质增效，推动一般贸易占比提高到40%以上，规上工业企业内销比例55%以上。力争用五年左右时间，先进制造业、高技术制造业增加值占比分别达到52%和50%，初步建成国内领先的创新型城市，在全省建设国家科技产业创新中心中走在前列，动能转换与经济转型取得根本性突破，实现东莞制造向东莞智造、东莞速度向东莞质量的跨越。市场主体超过100万户，其中既有3—5家超千亿的大型企业、500多家上市企业和新三板挂牌企业，也有大量创新型、高成长性的中小企业。智能手机、机器人及智能装备等超千亿元的新产业、新动能挑起大梁；新一代电子信息、半导体、新能源、高端装备、生物医药等产业形成气候；服装、家具、鞋业等传统制造业占据高端、不断焕发生机。先进制造业与现代服务业“双轮驱动”、外资和民营“比翼齐飞”、出口和内销“两分天下”的格局全面形成，东莞作为国际制造名城的地位得到进一步巩固和提升。

今后五年，要在更高起点上实现更高水平发展，必须大力实施城市品质提升计划，全面提高城市承载力与核心竞争力。东莞是一个组团式的现代化城市，在后工业化时代，这种特殊格局很好地顺应了高端产业和人才向往魅力小城和美丽乡村的追求，是我们参与区域竞争的重要优势和基础。但东莞也是一个地处穗港深经济走廊的典型通道城市，只有把城市品质和承载力提升上来，才能发挥自身优势，产生先进制造业“洼地效应”，与周边中心城市形成分工明确、联动发展的良好格局。否则，高端产业和人才过境而不留，优质要素反倒因周边中心城市“虹吸效应”而流失。我们必须强化“有什么样的城市、就有什么样的产业、就有什么样的人才”的理念，坚定“产城人”融合的方向，力争通过三五年时间的努力，实现城市品质内涵的跨越，全面提升城市承载力和竞争力。首先是推动城市总体格局大优化。完善一中心多组团的城市结构。通过功能轴线打造、重点单元建设提升和城市更新改造，适度强化城市中心和组团中心的集聚度，提升城市集约发展水平。因地制宜推动各组团片区打造一批以“产城人”融合为特征的魅力小城。推动一大批村（社区）打造成为设施完善、整洁有序、和谐宜居的美丽幸福村居。其次是促进城市基础设施大提升。抓紧推进地铁1号、2号等线路新建续建工程规划建设，配合推进穗莞深、佛莞惠等城际轨道以及赣深、深茂等铁路的规划建设，全面对接深圳、广州等城市的轨道交通，力争尽快形成网络。加快虎门二桥、深圳外环高速东莞段、莞番高速等建设，进一步完善“五纵四横六连”的高速路网。加快环莞快速路、桑茶快线等建设，形成“8”字形环城路网+多条放射状的市域快速路格局。加快打通与深圳、广州等城市及市内各镇街（园区）之间的断头路，大力推动东莞港建设，进一步强化内畅外联的交通网络。全面完善水、电、气、通信等基础设施网络。基本完成珠三角水资源配置工程东莞项目建设，科学推进江库联网工程，初步形成双水源保障新格局。第三是打好环境污染治理攻坚战。突出以水环境治理为核心，“十三五”完成不少于200条内河涌以及6公里海岸线的污染整治和生态修复，累计建成4500公里左右的截污管网，新建扩建15家集中污水处理厂及上百家分散式污水处理设施，基本形成厂网一体化运营管理的截污治水格局。主要河流基本消除劣五类水体，力争四个国考断面水质达到考核要求。空气优良天数比例达92%以上，土壤污染得到有效控制。新增生活垃圾实现无害化全焚烧零填埋处理，存量垃圾逐步消化处理，垃圾分类处理稳步推进。第四是再造宜居生态环境新品质。从满足市民高品质生活的需求出发，充分发挥生态本底好的优势，着力加强森林公园、湿地公园、体育公园、农业公园、滨海公园及现代渔港等建设，大力发展都市农业，切实完善城市配套，进一步彰显宜居生态之城的特色。力争经过三五年努力，体现东莞都市形象的大市区、体现魅力特色的各组团、体现基层活力的村容村貌都得到质的提升，“环莞一小时生活圈”基本实现，深莞惠3+2经济圈合作更加密切，东莞与穗港深一体化、同城化的态势更加明显。全市天更蓝、水更清，国际化、法治化、市场化的营商环境更加优越，对优质企业和高端人才的吸引力不断增强，东莞成为珠三角乃至全省全国最适宜创新创业的城市之一。

今后五年，要在更高起点上实现更高水平发展，必须坚持民生优先导向，让人民群众有更多的安全感、获得感和幸福感。把执政为民作为一切工作的出发点和落脚点，努力建设更加平安和谐、更加幸福美好的东莞。社会服务管理方面，突出以“智网工程”为主抓手，促进社会服务管理模式实现根本性变革，将网格化管理延伸到市域各个片区、拓展到社会治理各个层面，做到精准服务、精准管理、精准执法。加强社会治安综合治理，推进友善之城和平安文化建设，进一步提升群众安全感。强化公共安全和应急管理，全面排查整改各类安全隐患，妥善解决好群众的利益诉求，营造安定和谐的良好环境。公共服务配置方面，按照适度超前、循序渐进、实事求是的理念，提升优化公共服务的供给结构和质量，并根据组团格局优化资源配置，加大对次发达镇的扶持力度。建立健全非户籍常住人口与居住年限等条件相挂钩享受基本公共服务的机制，稳步推进基本公共服务均等化。民生事业发展方面，坚持每年办好十件实事，带动民生事业全面发展。着力打造东莞“慧教育”，提升各类教育优质均衡发展水平，加快高等教育发展，推动职业教育创新发展，打造教育现代化强市。深入推进“健康东莞”建设，积极构建与居民健康需求相匹配的医疗卫生服务体系。健全全民参保机制，建立更加完善的社保体系。构建轨道交通、常规公交、慢行交通一体化的公共交通体系，让市民出行更加便捷。提升城市文明方面，践行社会主义核心价值观，弘扬东莞城市精神，推进文化名城建设，全面提升市民素质和社会文明程度。把发展群众文化、群众体育作为基本方向，建设城市“十分钟健身圈”、镇村“十里文化圈”，承办好男篮世界杯、亚洲马拉松锦标赛，进一步打响篮球、莞香、音乐剧等品牌。深入改进作风，加强法治政府建设和廉政建设，营造风清气正、政通人和的良好环境。力争用五年时间，全市社会服务管理水平再上新台阶，“学有所教、劳有所得、病有所医、老有所养、住有所居”得到更好体现，在东莞工作生活的广大市民群众感到更加安全、舒适、开心和幸福。

2017年工作安排

2017年是实施“十三五”规划的重要一年，是推进供给侧结构性改革的深化之年，做好各项工作意义重大。今年政府工作的指导思想是：全面贯彻党的十八大和十八届三中、四中、五中、六中全会及中央经济工作会议精神，深入学习贯彻习近平总书记系列重要讲话精神，统筹推进“五位一体”总体布局，协调推进“四个全面”战略布局，自觉践行“五大发展理念”，按照省委十一届七次、八次全会和省委经济工作会议的要求，认真贯彻落实市第十四次党代会和市委经济工作会议的决策部署，坚持稳中求进工作总基调，以供给侧结构性改革为统领，以加速新旧动能转换为方向，以构建开放型经济新体制综合试点试验为契机，以“十大行动计划”为抓手，深化创新驱动，加快转型升级，强化执行落实，突出抓好实施倍增计划、加强招商引资、提升城市品质、优化民生保障等重点工作，促进经济平稳健康发展与社会和谐稳定，努力推动东莞在更高起点上实现更高水平发展。

今年全市经济社会发展的主要预期目标为：生产总值增长8—8.5%，人均生产总值增长8%左右，市一般公共预算收入增长9.5%，固定资产投资总额增长10%左右，社会消费品零售总额增长10%，进出口总额增长3%。先进制造业、高技术制造业增加值占比分别达到50%和40%，R&D比重提高至2.5%。全面完成年度节能减排目标任务。

围绕上述目标和要求，突出抓好以下工作：

一、加快创新驱动和转型升级步伐，打造智能制造新高地

以倍增计划带动集约化水平提升。按照“选好选优、培优培强”的原则，选取200家存量优势企业进行重点培育。支持企业通过科技创新、发展总部经济、推进兼并重组、加强产业链整合、强化资本运作等提升综合竞争力，力争用3—5年时间，推动试点企业实现规模和效益的倍增。同时，示范带动各镇街（园区）实施镇级倍增计划，引导全市存量优势企业全面提升产业集约、资源集约、空间集约水平。

实施精准服务企业行动。按“一企一策”方针，对龙头企业提供个性化服务方案，系统解决企业用地、人才保障房、员工子女入学、配套工厂落地及扩张等问题。深化“千干扶千企”服务，探索建立“企业十件实事”工作机制，帮助中小企业解决融资、引才等共性问题。积极探索解决市属转制企业历史遗留问题。清理规范行政审批中介服务事项。实行涉企收费目录清单管理制度，进一步减轻企业负担。

着力加强高企培育和创新平台建设。落实珠三角国家自主创新示范区总体实施方案。深入实施高企“育苗造林”行动计划，力争年内高企达2000家，高企后备企业1000家。鼓励高企组建重点实验室和工程中心，引导规上企业设立研发机构。以发展科技产业为重点，加大产业加速器环节的培育扶持力度，推动科研成果加速成为产能。加强新型研发机构绩效评估考核，加快大学创新城和高水平理工科大学建设。推进散裂中子源建设，力争9月出第一束试验束。启动建设先进高端材料研发实验基地。加快筹建名校研究生院，启动研究生实践基地建设。打造国家知识产权强市。

推进“机器换人”和智能化改造。实施智能制造示范工程，完成100条示范线建设。完善“机器换人”资金管理，用好省市融资租赁资金，撬动技改融资20亿元以上。集聚一批智能化改造系统集成商和服务商。引进培育一批龙头企业，推动机器人及智能装备产业产值增长20%以上。办好智博会与科技合作周。积极创建“中国制造2025”试点示范城市。

培育壮大智能终端等高端产业和新兴产业。构建完整的智能终端产业生态系统，力争成为全球智能终端产业最重要的研发设计、生产配套、品牌聚集基地之一。扶持新一代信息技术、新能源、生物医药、激光等产业加快发展。为1000家企业提供两化融合服务。大力推进“互联网+创新创业”。建设科技“四众”平台。支持企业利用互联网、大数据等创新商业模式，开展个性化定制、柔性化生产。大力发展研发设计、会展物流、文化创意和新型旅游等现代服务业。

促进科技金融产业深度融合。发挥产业投资母基金作用，形成“1+N”产业投资基金体系。推动企业上市和挂牌新三板融资，推动东莞上市板块扩容。支持企业通过发行公司债券、中期票据等筹集资金。支持金融机构拓宽业务领域，积极申报各类金融牌照。探索创建贸易金融创新示范基地，在松山湖等条件成熟的园区和镇街大力引进和发展基金业，促进股权投资基金、融资租赁、消费金融等新型经济金融组织集聚发展。力争私募投资基金突破250家。

大力弘扬企业家精神和工匠精神。强化“优秀企业家是经济发展脊梁”的理念，弘扬“厚德务实、敢为人先”的莞商精神，在全社会营造支持企业发展和推崇企业家精神的良好氛围。支持各大商协会及世界莞商联合会发展，推动企业家抱团发展。办好莞商学院，实施万名民营企业家培训计划。加强“创二代”的培养。鼓励有条件的民企引入现代管理模式。依法保护企业和企业家的合法权益。弘扬精益求精的工匠精神，实施技能人才培养行动计划，提升东莞制造品质。

二、加强重大产业项目建设招商，增强经济发展后劲

抓好年度225个重大项目建设。坚持市政府主要领导每月督导协调制度。完善重大项目全链条服务管理机制。推动项目审批提速，着力破解征地拆迁、管线迁改等难题。力争年内完成重大项目投资430亿元，其中重大产业项目290亿元。推动23个重大产业项目建成投产，新开工43个、总投资337亿元的重大项目。

主动到位加强产业精准招商。加强全市产业布局的统筹规划和引导，完善重大项目招商引资统筹流转和利益分享机制。实行引资、引智、引技有机结合，实施面向广深港澳台和欧美先进地区（国家）招商合作计划，瞄准100家左右高端制造项目开展精准招商。打造专业化招商团队，推动国际经贸合作网点建设，与投资促进机构、商协会建立常态化合作机制。对重点增资项目实行市镇领导挂钩督导，支持企业通过“工改工”扩建扩产。推动外地企业将在莞分支机构转为法人企业，鼓励企业在莞设立总部。力争引进超亿元内资项目100宗、超千万美元外资项目90宗。

强化产业用地统筹保障。做好基本农田与耕地调整工作，解决一批涉农土地历史遗留问题。分期用好新增建设用地指标，优先保障重大平台、重大项目、倍增计划、TOD开发的用地需求。加大对供而未用土地的处置力度，收回一批过期不动的项目用地指标。加强对镇街用地进度和产出效益的评估考核。处置闲置用地3000亩。探索农村集体土地使用制度改革，改进征地和拆迁补偿制度。完善镇村土地统筹利益分享机制。研究创新建筑工程招投标方式。开展被征地农民补偿款、征地留用地安置、农村土地“三乱”问题专项治理。加强土地收储整合，力争整合1000亩以上连片地块2—3块。

三、积极探索对外经贸合作新模式、新业态、新体制，促进开放型经济发展水平提升

加快构建开放型经济新体制综合试点试验工作。制定年度行动计划。在探索对接国际经济管理新模式等六大方面，推进20项重点任务，力争年底前取得阶段性突破。在经济管理服务改革等五大方面，推进32项具体改革措施，力争形成一批可复制推广的改革经验。积极向上争取第一批19项支持政策。

大力推动加工贸易创新发展。制定加工贸易转型升级评价体系。建立鼓励加工贸易企业技术创新、品牌推广、营销培育、质量提升的四大机制。扶持港台企业转型升级。办好加博会和台博会。鼓励加工贸易企业在莞设立地区总部或与民营企业融合发展。推广以企业为单元的加工贸易监管模式。搭建“东莞制造”品牌推广服务平台，推动优势企业联合“走出去”。

深度对接国家“一带一路”战略。加快建设广东（石龙）铁路国际物流基地，常态化开通中亚、中欧国际班列。推动华南火车邮件物流枢纽建设。推进中俄贸易产业园先行启动区建设。实施“一港一铁”、“多式联运”工程，推动东莞港与日韩和东南亚国家沿线港口建立直航航线。探索创建滨海湾“一带一路”国际合作示范区。办好海丝博览会。推进广东海丝跨境商品展示交易中心、南非商品中国（东莞）展示交易中心、巴西中国经济贸易促进会总部基地、广东（东莞）珠宝玉石交易中心等建设。

大力发展跨境电商和保税物流。实施电子商务企业50强清单行动计划，抓好物流标准化等四大服务平台建设。加快建设电子商务统计系统。积极推动跨境电商通关模式改革。加快东莞跨境电商中心园区、京东现代服务业产业园、珠三角汽车博览中心等项目建设。建成东莞国际邮件互换局兼交换站。对接香港“超级中国干线”业务，为企业提供空陆多式联运服务。推进全国外贸综合服务企业试点。力争综合保税区早日获批。

积极参与粤港澳大湾区建设，加强区域协作与对口帮扶。主动对接广东自贸区，推广出口退税无纸化等37项改革经验。推进深莞惠3+2经济圈建设，加强与深圳、广州在交通对接、产业互动、协同创新、环境治理等层面的全方位合作。打造莞港澳台创新创业基地。积极争取将水乡经济区、滨海湾开发区和东莞港纳入粤港澳大湾区核心区域重大平台。扎实推进对韶关、揭阳帮扶工作。落实援藏援疆任务，抓好巴宜区富民兴藏工作，推进兵团草湖工业园示范项目。深入开展与昭通市的扶贫协作。加强与四川雅江县、九龙县和重庆巫山县的对口联系。

四、构建园镇统筹发展新格局，推动区域协调发展

推动松山湖、水乡管委会与周边镇联动协同发展。用好松山湖珠三角国家自主创新示范区政策，加速集聚创新要素，增强松山湖辐射带动能力。进一步明确水乡管委会职能定位，支持设立控股平台和开发基金，加快水乡新城和重大基础设施建设。加大松山湖、水乡管委会与周边镇的统筹发展力度，实施市级行政审批事项服务前移，高标准开发建设片区重点区域，探索建立园镇统筹发展的决策、规划、招商、投融资、项目布局等机制。启动一批一体化的交通、环境工程建设。

创新园区开发模式，“一园一策”加快园区发展。明确市与园区及相关统筹发展镇街的事权划分，加大市级权限下放园区力度。着力解决制约园区发展的行政管理、土地开发、投融资、交通连通等共性问题。理顺银瓶创新区政企合作关系，抓好基础设施和公共服务项目建设，加快粤海产业园和银山科技园建设与招商。推动东莞港发展，加快疏港交通快速通道建设，打造立沙岛、主港区、新沙南三大临港产业板块。加快滨海湾开发区建设，科学谋划发展定位，完成5000亩以上滩涂填土，加快围填海和基础设施建设，探索推进土地1.5级开发。启动原东部工业园企石辖区开发，解决土地遗留问题，建立利益平衡和投融资机制。

扶持镇村经济发展。推动专业镇从“一镇一品”向“一镇一品一特色服务”提升，促进五金模具、纺织服装等产业集群向价值链高端延伸。推进特色小镇建设。设立10亿元的扶持次发达镇产业发展资金池。用好三年6.4亿元市内帮扶资金，力争年内70%的次发达村经营性纯收入达到300万元。探索搭建集体经济与实业投资、金融信托等机构的合作平台，推动集体经济从单一的物业租赁向物业型、服务型和投资型多元化发展转变，探索建立“效益决定分配、盈利共享、风险共担、可增可减”的股东分红机制。

五、全面启动新一轮基础设施及城市配套规划建设，进一步提升城市品质和内涵

以强化中心城区功能为先导，统筹推进市域顶层设计。抓住土地利用总体规划调整完善、国家住建部城市总体规划改革试点等契机，推进“多规融合”，力争中心城区土规、城规重合率达90%以上。探索组团规划编制和控规编制改革，开展城市密度分区和容积率专题研究，加强对中心城区、松山湖核心片区等开发强度的控制和引导。推进南城国际商务区等重点单元规划建设，探索对同沙生态公园周边片区、东江大桥至环城西路的滨水片区实施综合规划开发，研究推动东莞水道、厚街水道、汾溪河的综合改造。研究松山湖大道、环城路、东部快速路、新城大道、生态园大道等道路连接体系的综合改造提升。启动市中心主干道交通改造工程，新建和打通支线道路，研究缓解市区交通拥堵的系统改造和治理方案。

加快轨道交通等重大基础设施建设。推进赣深客专东莞段建设。加快穗莞深、佛莞惠等城际轨道和地铁1号线建设。加快1号线南延线（对接深圳6号线支线）、西延线（对接广州5号线），2号线三期及南延线（对接深圳20号线），以及深圳13号线北延至松山湖的前期工作。优化协调莞番高速线位，加快虎门二桥、莞番高速、深圳外环高速东莞段、常虎高速虎门港支线二期、桑茶快线及延长线、中洪支线等建设改造。建成松山湖大道大朗段。推进环莞快速路建设，力争年内二期建成通车，加快推进三期建设。研究推动市区至松山湖第二通道的规划建设。加快疏港大道延长线建设，启动望沙路升级改造、水乡地区横向通道工程建设。推进东莞站配套工程、新沙港区二期工程等建设。试点推进海绵城市和地下综合管廊建设。推进信息基础设施建设大会战。启动电网升级行动。

实施打通断头路行动计划。建成梨川大桥、东宝河新安大桥。加强穗深莞惠交通对接，加快东平东江大桥、龙江东江大桥、金龙路、恒心路、黄江生态路等建设。加快市内道路连通，抓好石龙南岸二桥改建、道滘大桥重建等工程。系统研究市际、市内、镇村等一批断头路的贯通方案，逐步启动实施。

加快城市更新连片改造和TOD开发建设。完善统筹协调城市更新工作的体制机制。出台连片改造专项政策。建立城市更新成效与新增用地指标分配挂钩制度。加快东城黄旗南、万江龙湾滨江等5个共约1.4万亩的片区改造。新启动南城东华、长安科技商务区等8个共约3万亩的片区改造。支持东城“33小镇”、鳒鱼洲、常平科技加速园、黄江裕元灵狮小镇等旧工业区活化更新。探索推进挂影洲围的统筹开发。每个次发达镇至少实施1个城市更新项目，其他镇街实施2—3个。启动虎门站、东莞西站、东莞火车站、东莞东站、松山湖北站、黄江北站等TOD开发试点，示范带动全市TOD开发。

推进魅力小城和美丽幸福村居建设。出台实施魅力小城建设三年行动计划，选择若干片区重点单元作为示范试点，切实加强产业指引、空间规划和资金支持，推进一批规划建设有品质、产业发展有特色的魅力小城建设。启动美丽乡村特色连片示范建设。力争完成100个村（社区）美丽幸福村居建设。推动麻涌、樟木头、清溪等镇创建省级全域旅游示范区。

按照“建管结合、重在管理”的思路，大力整治交通拥堵问题。完善多部门联动的交通工作机制。优先发展公共交通。完善提升枢纽站、换乘站的规划建设。做好轨道交通站点与公交的衔接。优化公交线路、班次和服务水平。试行政府购买公交服务运营模式。出台网约车管理细则。研究通过增设掉头立交等微治理手段，对交通拥堵路段实施系统改造。建设智能交通管理系统，提高交通管理信息化水平。

加强城市精细化管理。深入推进城市执法体制改革。建立城乡环境“六整治”长效机制。出台户外广告设置、市容环境卫生管理等法规规章，建全城市管理考核评价机制。严格控制新增“两违”。对历史遗留违法建筑摸清底数、建立台账，探索实施分类处理，修订产权补办手续办法，逐步消化存量违法建筑。

深化投融资体制改革。编制中长期政府投融资规划。完善政府债务管理。推广应用PPP模式，探索政府购买服务。创新交通、环保、园区开发、民生项目的投融资模式。优化整合市属金融企业股权。进一步增强市属国有企业的融资能力。梳理盘活各类国有和集体闲置资产。深化市属国资国企改革，推进国有资产重组，增强在公益性、战略性领域的支撑保障作用。

六、加强污染治理和生态绿化，提升生态文明建设水平

全面打响水环境污染治理攻坚战。年内新建不少于500公里、两年新建1800公里截污次支管网，年内新建扩建10家集中污水处理厂。启动分散式污水处理设施建设。落实四大重点流域河长制。加快茅洲河、石马河等跨界河流的综合治理。“一河一策”加快内河涌整治，确保两年内完成不少于100条内河涌污染整治和生态修复。完成年度黑臭水体达标整治任务。推进饮用水源保护工程建设。

加强固废、大气、土壤污染治理。完成麻涌、横沥、市区环保热电厂新建扩建工程，将日处理能力提升到1.2万吨，实现新增生活垃圾全部无害化焚烧处理。建成麻涌餐厨垃圾处理项目和谢岗飞灰填埋场项目。加快海心沙循环经济产业园和建筑垃圾堆场建设。做好绿色供应链环境管理试点工作。全面淘汰黄标车。加强臭氧污染防治，开展重点行业挥发性有机物污染整治。推进燃煤工业锅炉整治和沙角电厂排放改造。加快中堂造纸基地等一批集中供热项目建设。开展土壤环境质量调查，推进3个土壤污染修复治理试点，形成经验推广至全市。

实施森林公园分类提升工程。对大岭山等森林公园进行升级改造，启动银瓶山三期建设，完成清溪湖森林通道等项目。做好城市森林公园周边空间的开发利用、郊野森林公园周边旅游设施的规划建设。加强“小山小湖”保护与开发。推进同沙湿地公园、黄江农业公园等建设，建成植物园一期工程。推进水源涵养林、生物防火林带等改造和营建。加快森林小镇建设。

七、推进社会治理现代化，营造安全稳定的社会环境

全面推开“智网工程”。完成网格员队伍整编和指挥调度中心建设，加强队伍培训，建立科学的日常管理、考核奖惩制度。全面启用“智网工程”信息系统。推动基层市场网格化监管等更多服务管理事项入格，推动大数据在社会治理中的创新运用，做到横向打破信息孤岛、纵向联动政府社会，实现精准管理。

全面强化社会治安管理。重拳打击电信诈骗、网络金融犯罪、“两抢一盗”、黑恶势力等违法犯罪行为，加快建设立体化社会治安防控体系，推动社会治安实现更加明显的好转。深入开展“以案说防”活动，大力推动“全民创安”。加快公安情报指挥中心升级改造。加强和改进公安派出所工作。推动出租屋管理地方立法。完善通过法定途径分类处理信访投诉的机制。创新基层劳资治理机制，实行兼职仲裁员办案制度。加强第三方调解组织建设，完善诉前联调机制，更好地发挥社会力量在管理社会事务中的作用。

全面加强安全生产、消防安全、食品安全等工作。推动“智慧安监”二期建设，加强基层专职安全员队伍建设。狠抓危化行业安全监管，推进立沙岛整岛封闭式管理，加快建设危化品应急救援基地。深入开展城市风险点、危险源排查整治行动和建筑、轨道交通施工安全大检查。实施部门“分镇包干”督导消防工作制度，加强消防安全专项治理，抓好省市挂牌督办重点镇村的集中整治。加强灾害性天气安全防范。打造50家食品安全示范市场，新建150家农贸市场快速检测室，建成市食品药品检测中心，铁腕查处食品药品安全违法行为。落实粮食保障任务。建设以企业信用管理为核心的科学市场监管体系。加强应急预案管理和演练，提升突发事件应对能力。

八、优化公共服务供给，切实增进民生福祉

努力提供更多的优质教育资源。编制各镇街、园区公办学校建设规划，制定居住区配套教育设施规划建设管理办法，新建扩建21所公办学校。实现民办义务教育标准化学校基本全覆盖、优质学校比例提升到60%。大力发展公益普惠性幼儿园。推进高等教育发展。优化积分制入学办法。完善企业人才子女入学机制。购买民办义务教育学位同比增加20%。创新和推广委托管理等办学模式，完善学校绩效管理机制。推进教育信息化和“慕课”试点。引进德国、台湾等优秀职教教学团队，提升办学水平。争取成功创建省现代职业教育综合改革示范市。

全面推进卫生强市建设。优化整合医疗资源，推动区域医疗中心建设。加快“一中心三平台”信息化建设。提升社区卫生服务机构水平，完善分级诊疗制度，力争基层诊疗服务量占65%以上。建设市第九人民医院。鼓励市镇医院联合办医，推动部分公立医院向专科医院、医养结合机构或社区卫生服务中心转型。引进一批高层次医疗卫生人才，加强“东莞名医”和省名中医药专家培养，推进中医药强市建设。

促进房地产平稳健康发展，建立健全住房保障体系。落实房地产调控和住房限购政策。稳定房地产市场土地供应。加快非住宅商品房去库存。探索建立房地产用地招拍挂的创新机制。研究出台有利于长远发展包括保障房、人才房在内的、购租并举的住房保障体系政策。研究改善刚需部分村（居）民居住条件的政策。推进不动产统一登记，建立完善产权保护制度。出台物业管理办法。稳步扩大住房公积金缴存覆盖面。

着力加强文化体育建设和民生保障。推进基层综合性文化服务中心试点建设。实施“文物保护与利用”和“城市历史文化特色与价值强化”两大工程。挖掘工业遗产资源，推进文化创意改造。争取成功申报国家历史文化名城。争创全国版权示范城市。推动全民健身运动，办好亚洲马拉松锦标赛和市运会。继续办好十件实事。将低保标准从每人每月610元提高到720元。推广电子社保建设。新建25个社区综合服务中心。落实“8+1”社会救助制度。发展志愿服务、慈善、老龄和残疾人事业，新增养老床位500张以上，开展医养结合试点。加快市老年大学新校区建设。做好关心下一代工作，积极帮助青少年成长成才。开展新一轮双拥模范城创建。扩大法律援助范围。实施“巾帼关爱行动”。落实就业创业扶持政策。

九、强化法治意识和担当意识，提升与更高水平发展相适应的行政服务和履职能力

加强法治政府和廉洁政府建设。依法接受人大及其常委会的监督，自觉接受政协的民主监督，接受社会和舆论监督。完善政府法律顾问、经济顾问和决策咨询机制。全面公开部门预决算和“三公”经费支出信息，推动镇街公开财政预决算信息。加强地方立法工作，实施“七五”普法规划，深入开展法治创建活动。扎实推进反腐倡廉建设。

深化“一门式一网式”改革。组建市政务服务办和镇街（园区）政务服务中心。加快市镇实体办事大厅和网上统一申办受理平台建设，实现“一窗通办”、“一网通办”。深化项目投资管理体制改革，推进以承诺制为核心的项目直接落地改革。完善商改“宽进严管”体系。加快行政审批标准化建设，优化办事流程，推进数据共享和信息互认，让市民逐渐从“排队办事”变为“手机办事”、从“群众跑腿”变为“数据跑腿”。

提振敢于担当、主动作为的精气神。摒弃小富即安、“顺其自然”的工作心态，增强干事创业、克难奋进的攻坚锐气。加强工作督促考核，宽容改革创新失误，营造鼓励干事、支持改革、奖优罚劣的良好政风环境，进一步提振干部勇于担责、主动作为的精气神，务求高质高效完成全年目标任务。

各位代表！新的征程已经开启，新的目标催人奋进。让我们紧密团结在以习近平同志为核心的党中央周围，在省委、省政府的正确领导下，在市委的坚强领导下，与全市人民一道，务实创新、真抓实干、团结奋进，为推动东莞在更高起点上实现更高水平发展而不懈努力，以优异成绩迎接党的十九大胜利召开！
